# Potential novel risk factors for autochthonous and sylvatic transmission of human Chagas disease in the United States

**DOI:** 10.1186/1756-3305-7-311

**Published:** 2014-07-04

**Authors:** Melissa N Garcia, Peter J Hotez, Kristy O Murray

**Affiliations:** 1 Section of Pediatric Tropical Medicine, Department of Pediatrics, National School of Tropical Medicine, Baylor College of Medicine and Texas Children’s Hospital, 1102 Bates Avenue #550, Houston, Texas 77030, USA

**Keywords:** Chagas disease, Hunting, United States, Transmission, *Trypanosoma cruzi*, Wildlife reservoirs, *Triatoma*

## Abstract

Chagas disease is an emerging vector-borne disease in the United States that causes progressive dilated cardiomyopathy in a third of infected humans. While transmission studies have been performed in Latin America, little is known about the source of infection in locally acquired cases in the United States. This letter describes the underlying factors possibly leading to an increased risk of disease transmission among high-risk groups in the United States.

## Findings

Chagas disease is a vector-borne disease caused by infection with the parasite *Trypanosoma cruzi* (*T. cruzi*). There are 11 established *Triatoma* insect vectors reported in the United States that can transmit *T. cruzi* to a large number of competent mammalian hosts [1,2]. These *Triatoma* insect vectors typically infest mammalian dens and burrows resulting in sustained interaction between vector and mammalian host [3]. Recent evidence of establishing sylvatic transmission cycles have been increasingly reported between vectors and mammalian hosts in the southern United States [3].

Rare reports of locally-acquired human cases have been reported in Texas and adjacent southern states [4]. This number is believed to be an underestimation due to disease underreporting, lack of active surveillance programs, and minimal physician awareness about the disease [5]. With the recent recognition of Chagas disease transmission in the US, it is critical that we understand how human transmission is occurring for the development of effective intervention studies. Although there is reason to believe domestic transmission from kissing bugs would most likely occur in areas of extreme poverty and dilapidated housing in the southern US, a second unexplored risk for disease transmission could be occurring as a result of spillover in a sylvatic transmission setting.

Over this past year in Houston, we began enrolling a cohort of presumed healthy people who were found positive for Chagas disease through the blood donor-screening program at the regional blood center (approved by the Institutional Review Boards at Baylor College of Medicine and the Gulf Coast Regional Blood Center). While we are still in the process of enrollment, we were astonished by the higher than expected percentage (6/17; 36%) of people who acquired their infection here in the US as opposed to a Chagas endemic Latin American country. Interestingly, most (67%) of these individuals reported an extensive history (10+ years) of hunting or camping activities, leading us to speculate that time outdoors in rural settings could place individuals at higher risk for infection.

In the United States, 90 million people take part in hunting, fishing and wildlife associated recreation [6]. It is plausible that these activities increase one’s chance of disease transmission by increasing their exposure to both the vector insect and possibly to the parasite directly. Game hunters typically use tree stands and hunting blinds during early morning hours, which expose them to vectors without protection. Hunters and fishermen spend an average of 536 hours per year outside compared to 116 hours for the average American [6,7]. In our study, all of those with a history of hunting reported camping in a tent and/or sleeping outside without shelter. Sleeping outdoors without adequate shelter or in *Triatoma-* infested structures could place these individuals at higher risk for vector exposure, especially at night when vectors seek a blood meal.

In addition to vector exposure during outdoor activities, we think it is likely that a hunter could be exposed to the parasite through direct contact with infectious blood and tissues of wild animal reservoirs. Triatomine insects peak in abundance between May and July when temperatures are ideal for night flying (20°C) [8]. Naïve wildlife is likely infected at this time due to vector abundance. During acute infection, parasitemic wildlife could expose hunters to highly infectious blood and tissue during skinning and field dressing activities [4]. In our pilot study, no one reported wearing gloves during the process of skinning, further exacerbating their potential for blood borne pathogen exposure. Blood-borne transmission is a known mechanism for human-to-human infection, but has never been reported in this particular setting.

In the southern United States, there are twenty-four wildlife species that have been demonstrated as important hosts in the maintenance of the sylvatic transmission cycle [3]. In Texas, six of these twenty-four species have tested positive for *T. cruzi* and are permitted to be hunted all year long by the Texas Parks and Wildlife Association [3,9]. These six species include raccoon, opossum, striped skunk, nine-banded armadillo, American badger, and coyote. With up to 40% of wildlife positive for *T. cruzi *[10,11], blood-borne infection through breaks in the skin of hands during the skinning and dressing process poses a reasonable risk to hunters in the US.

The purpose of this letter is to hypothesize novel risk factors for Chagas infection in the US, and we feel these hypotheses warrant further investigation. With Chagas disease emerging in the United States, it is imperative that we understand the transmission dynamics for the prevention of infection. Seroprevalence studies in these potentially high risk as well as normal control groups would enable us to understand the relevance of our early findings. Clinicians and public health officials should consider screening for *T. cruzi* in those presenting with cardiac disease, particularly dilated cardiomyopathy, and performing case investigations to identify their specific source(s) of infection.

## Competing interests

The authors declare that they have no competing interests.

## Authors’ contribution

MNG, PJH and KOM conceived of the study. MNG performed the analysis and wrote the manuscript. All authors read and approved the final manuscript.
